# How to Train Your Dragon: Harnessing Gamma Delta T Cells Antiviral Functions and Trained Immunity in a Pandemic Era

**DOI:** 10.3389/fimmu.2021.666983

**Published:** 2021-03-29

**Authors:** Jonathan Caron, Laura Alice Ridgley, Mark Bodman-Smith

**Affiliations:** Infection and Immunity Research Institute, St. George’s University of London, London, United Kingdom

**Keywords:** gamma delta T cell, innate immunity, trained immunity, antiviral, virus, COVID-19, BCG, vaccine

## Abstract

The emergence of viruses with pandemic potential such as the SARS-CoV-2 coronavirus causing COVID-19 poses a global health challenge. There is remarkable progress in vaccine technology in response to this threat, but their design often overlooks the innate arm of immunity. Gamma Delta (γδ) T cells are a subset of T cells with unique features that gives them a key role in the innate immune response to a variety of homeostatic alterations, from cancer to microbial infections. In the context of viral infection, a growing body of evidence shows that γδ T cells are particularly equipped for early virus detection, which triggers their subsequent activation, expansion and the fast deployment of antiviral functions such as direct cytotoxic pathways, secretion of cytokines, recruitment and activation of other immune cells and mobilization of a trained immunity memory program. As such, γδ T cells represent an attractive target to stimulate for a rapid and effective resolution of viral infections. Here, we review the known aspects of γδ T cells that make them crucial component of the immune response to viruses, and the ways that their antiviral potential can be harnessed to prevent or treat viral infection.

## Introduction

It’s estimated that on average, a human being will be infected with about 10 different viral species over a lifetime (1), including influenza viruses, coronaviruses, noroviruses and rhinoviruses. Most of these viral infections result in either no disease or mild symptoms, and viral clearance in a matter of days or weeks. However, the increasing emergence of new viruses, to which human populations have no existing immunity, raises the potential for pandemics posing a threat to global human health that needs to be addressed.

During a viral infection, the successive and functional cooperation of the innate and adaptive immune systems is crucial in order to control the viral load and lead to a successful resolution of disease. The early detection and reaction by the immune system to viral infection is fundamental for the subsequent course of infection. This early response includes the production of cytokines and cytotoxic factors by first-line innate effector cells including macrophages, neutrophils, natural killer cells and Gamma Delta (γδ) T cells. This early ‘innate’ arm of the immune system also begins to recruit the adaptive arm to tailor the response and lead to immune memory. γδ T cells in particular are of the utmost importance as their large numbers in tissues, their pre-activated phenotype and rapidity of response make them a central player in the fight against viruses (2). They represent 1-5% of blood lymphocytes and constitute between 10–100% of T cells in “barrier” sites such as lung, gut and skin (3). γδ T cells migrate to these organs during early development and persist there as resident cells (4) with non-redundant features of surveillance compared to the other tissue-resident lymphocytes (5, 6). In addition, γδ T cells acquire a pre-activated phenotype early in their development that allows the rapid induction of effector functions upon detecting cellular stress and infection. Indeed, γδ T cells have been shown to be one of the first immune cells to react to viral entry (7). The importance of γδ T cells for an efficient antiviral response is illustrated by γδ T cell-deficient mice which show severely impaired responses to both primary and secondary infection (8, 9). These mice also demonstrate substantial increases in viral titers immediately post-infection as well as increased mortality compared with control mice. The precise mechanisms deployed by human γδ T cells against viruses are still incompletely understood, but their ability in early sensing of infection, quick activation and cytotoxicity against a wide array of viruses, including cytomegalovirus (CMV), influenza A virus, hepatitis B (HBV) and C (HCV) virus, human immunodeficiency virus (HIV) and severe acute respiratory syndrome-related coronavirus (SARS-CoV), has triggered interest in a better definition of these under-studied lymphocytes and in ways of harnessing their potential for therapies (2). This review aims to provide an insight into γδ T cells’ protective functions in human pathologies and to illustrate the necessity of including innate immunity in the design of antiviral strategies.

## Sensing Viruses: γδ T-Cells as Early Responders

Despite their active roles in many human infectious diseases, the pathways used by γδ T cells to sense pathogens and initiate rapid responses remain largely unknown. In this section, we will explore some of the principal signals that are critical for γδ-T cell-mediated antiviral activity.

### Toll-Like Receptors

In addition to their strategic position, γδ T cells express a diversity of receptors for sensing both viral particles directly and infected cells. Firstly, the presence on γδ T cells of both membrane expressed and intracellular pattern recognition receptors (PRRs), which bind conserved pathogen-associated molecular patterns (PAMPs), is a major tool for virus detection. Of particular importance are Toll-like receptors (TLRs) that respond independently of any other receptors to stimulation by virus-derived molecules.

TLRs are expressed on the cell membrane, where they can directly recognize PAMPs like viral glycoproteins and glycolipids (TLR2 & 4) (10–12). They are also present on endosomes and lysosomes where they detect viral single-stranded (TLR7) and double-stranded (TLR3) RNA (13), as well as CpG nucleotides (TLR9) present in the extracellular environment or produced during intracellular replication of many viruses. All TLRs (but TLR8) are expressed on γδ T cells in peripheral blood of human donors (14), and they are quickly upregulated during activation (e.g. by TCR stimulation) (15).

The binding of viral ligands to TLRs leads to the activation of several transcription factors such as interferon regulatory factor 3, 5, and 7 (IRFs) and nuclear factor-κB (NF-κB) (16). This activation induces an antiviral program, including production of interferons, pro-inflammatory cytokines (IL-1, TNF-α) and other associated molecules. Through positive feedback processes, interferons are able to enhance many TLRs (17).

### Natural Killer Type Receptors

In addition to PRRs, γδ T cells also express several other receptors that mediate their optimal activation during viral infection, by directly triggering their own signaling effect, and/or modulating TCR signaling. Among these are NK type receptors (NKRs) including natural killer group 2-member D (NKG2D), DNAX Accessory Molecule-1 (DNAM1) and the Natural Cytotoxicity receptors (NCRs) NKp30, NKp44 and NKp46.

The activating NKG2D molecule is an important stimulatory receptor expressed on γδ T cells which provides a critical role in stress antigen recognition (18). In humans, the ligands of NKG2D have been identified as stress‐inducible MHC class I related molecules A/B (MICA/MICB) and members of the UL16-binding protein family (ULBPs) (19). These molecules have been shown to be upregulated in response to stress, including viral infection. For example, during CMV infection of fibroblasts, MICA and ULBP1-3 have been shown to be upregulated (20). MICB is induced in macrophages infected by influenza A or Sendai virus (21). CD4+ lymphocytes infected by HIV also display an upregulation in ULBP1-3 (22). Furthermore, MICA, MICB and ULBP4 have been shown to be upregulated in response to Epstein-Barr virus (EBV) infection allowing activation of γδ T cells (23, 24). Recognition of these ligands induces signaling through NKG2D and rapid Ca2+ responses, triggering protein kinase C (PKC)-dependent co-stimulation of the TCR (25), but can also signal independently of TCR signaling (18). Blockade of NKG2D but not TCR resulted in decreased killing suggesting that recognition is principally mediated by NKG2D, and activation achieved through TCR (26). Ligand recognition might actually involve the two receptors, as ULBPs have been suggested to engage both NKG2D and Vγ9Vδ2 TCR (24). Alternatively, the binding of TCR and NKG2D to MICA has been reported to be mutually exclusive, with a dynamic influenced by the higher affinity for the latter (27).

DNAM1 or CD226 is another NKR involved in γδ T cell activation. It is expressed at a low level constitutively and is upregulated following stimulation of the cell (28). The ligands of this receptor include poliovirus receptor PVR (CD155) and nectin-2 (CD112), key receptors that play a role in viral entry and have been shown to be upregulated in response to cellular stress such as infection by viruses including CMV, HIV, EBV (29–31). Interaction of DNAM1 with its ligands triggers γδ T cell effector functions, notably cytolytic granule exocytosis and interferon-gamma (IFN-γ) production against tumors (28), but more studies are needed to establish if it has similar effects during a viral infection.

Finally, γδ T cells have been shown to express members of the NCR family, including NKp30, NKp44 and NKp46. These receptors were originally documented on NK cells and were shown to coordinate cytotoxic responses against tumor and infected cells. They play a key role in infection by CMV, as infected cells express NKp30 ligand B7-H6 (32). NKp44 and NKp46 bind hemagglutinin (HA) present on influenza (33, 34) and vaccinia viruses (35) as well as hemagglutinin-neuraminidase (HN) on Newcastle disease virus (NDV) (36). Numerous other pathogens such as West Nile and dengue viruses have also been shown to bind these receptors *via* unidentified proteins (37). While not expressed constitutively on γδ T cells, studies have shown that the expression of NCRs can be induced following activation (38). NCRs are instrumental for γδ T cells antiviral function, as shown for example in the case of HIV suppression *via* NKp30-dependent activation of γδ T cells (39), or cytotoxicity inhibition by specific blockade of NKp44 (40). These receptors have been shown to mediate granzyme B production and cytotoxicity in a TCR-independent manner (38).

### T-Cell Receptor

Gamma delta T cells are also capable of responding to infected cells *via* their T-Cell Receptor (TCR). The TCR recognition of γδ T cells is independent of MHC restrictions (41) and has been shown to bind to a variety of non-processed antigens (42) including MHC-like molecules (43), HSPs (44) and HSP-regulated proteins (45), several glycoproteins, lipoproteins and phosphoantigens (pAg) (46). Many of these antigens are upregulated in an infectious context, as shown earlier for MICA and MICB, and γδ T cells rely on them for optimal activation and antiviral function, as exemplified by the correlation between pAg synthesis of EBV- or influenza A-infected cells and γδ T cells cytotoxicity against them (47, 48). The role of the γδ TCR is illustrated by blocking studies, resulting in the loss of recognition, for example in CMV-infected cells (49). Conversely, transferring TCR from a CMV-reactive clone to a TCR-deficient cell line is sufficient to confer reactivity against CMV-infected targets (50).

In humans, γδ T cells can be classified into two main populations according to their TCR expression: Vδ1 and Vδ2 γδ T cells (51). Vδ1 γδ T cells are generally resident lymphocytes, abundant in mucosal surfaces and epithelia of the digestive, respiratory and urogenital tracts; in contrast, Vδ2 γδ T cells are circulating lymphocytes and constitute the majority of peripheral blood γδ T cells (52). There is some evidence to suggests that the tissue specificity of γδ T cells is shaped by the selective activation resulting from the interaction between the TCR and a family of presenting molecules called butyrophilins (BTN) and butyrophilins-like proteins (BTNL) (53, 54).

Vδ1 γδ T cells proliferate during some chronic viral infections, including HCV and HIV (55, 56). They display antiviral potential with the production of T-helper cell type 1 cytokines (57) and direct cytotoxicity toward infected cells (58). Similarly, activation and proliferation of Vδ2 γδ T cells have also been shown to be increased early during the acute phase of many viral infections. These cells can display potent antiviral responses and mainly recognize pAg synthesized by infected cells *via* the interaction between their TCR and the BTN3A1 (CD277) presenting molecule (59, 60). This activating signal is capable of stimulating Vδ2 γδ T cells independently of the virus type (48).

Activation of γδ T cells by the integrated signals from the PRRs, NKRs and TCRs induce an antiviral state characterized by proliferation and phenotypic specialization. Indeed, as seen for example in hepatitis C virus (HCV) patients (2), during infection by herpes simplex virus (HSV) (61), or following an encounter with EBV (62, 63), there is a rapid proliferation of γδ T cells seen in the blood where they can expand from approximately 1% of circulating T cells in steady-state to over 50% following viral infection. These expanded γδ T cells express activation markers like CD69, CD38 and HLA-DR absent in healthy individuals (64, 65), but also effector molecules such as perforin, granzymes, granulysin contained in cytolytic granules and FasL or TRAIL.

## WHODUNNIT: γδ T-Cells as Virus Killers

The strategic position of γδ T cells for immune surveillance, and their capacity to recognize a unique and wide array of danger signals allows them to rapidly detect viral infection. This activation generates a high number of functionally active cells, ready to deploy their full antiviral potential *via* multiple routes, either direct killing of infected cells or indirect inhibition through production of noncytolytic factors and interactions with other components of the immune system.

### Direct Antiviral Action

γδ T cell-mediated direct cytotoxicity is executed by diverse pathways, including secretion of cytotoxic mediators stored in granules such as perforin (66), granzymes (67, 68) and granulysin (69) and expression of members of the death-inducing TNF family of ligands and receptors, including tumor-necrosis factor-related apoptosis-inducing ligand (TRAIL) (70) and FasL.

γδ T cells uniformly express abundant perforin, granzymes and granulysin in their cytoplasmic granules (71–74) and are able to degranulate after specific recognition of virus-infected cells (75). Interestingly, the granules’ content varies with cell type and immunological context, influencing the outcome. For example, Granzyme M, which is highly expressed by γδ T cells, is regulated differently than Granzyme B and initiates a unique cell death pathway independent of caspase activation (76, 77). In addition to the induced apoptosis of infected cells, Granzyme M also directly inhibits viral replication by cleavage of essential virus proteins (78). Similarly, γδ T cell granules contain Granzyme H and K which have various antiviral activity against adenoviruses, Influenza virus, HBV and HCV (68, 79–82).

Despite the central role of the cytolytic granules in immune-induced apoptosis, several observations of target cell death in the absence of Ca2+, perforin, or granule exocytosis suggests the existence of alternative pathways of cytotoxicity. The FasL-Fas pathway is such an alternative mechanism of direct killing used by γδ T cells (83). Fas is induced in the membrane of virally infected cells (84) and binds to FasL expressed on γδ T cells. This leads to caspases activation and apoptosis in a manner not dissimilar to the one triggered by Granzyme B (85). γδ T cells upregulate FasL as early as 1 hour after stimulation (via NF-kB), and are capable of keeping a high and sustained expression during an immune response (86).

### Indirect Antiviral Actions

Mounting evidence indicates that γδ T cells also exert their protective function in the elimination of pathogens by producing cytokines, chemokines, and interacting with other components of the immune system.

During a viral infection, targeted cells can produce cytokines like TNF-α, IL-1, IL-6, IL-18 (87) which participate in the activation of γδ T cells both *in situ* and in the peripheral blood. During activation, these γδ T cells upregulate the chemokine receptors CXCR3/5, and CCR1/5, allowing additional recruitment to the site of inflammation, rich in CCL3/4/5 and CXCL9/10/11 [86–88].

Within a few hours of activation, γδ T cells release high amounts of cytokines, among which is IFN-γ, a key antiviral molecule capable of suppressing viral replication as well as recruiting and activating complementary immune cells like NK, macrophage or killer T cells. *In vitro*, the non-cytolytic antiviral activity of IFN-γ has been demonstrated in infections with hepatitis viruses (HBV & HCV), herpesviruses, orthopoxviruses, picornaviruses, retroviruses, influenza and others (88). IFN-γ induces the transcription of several genes called Interferon-Stimulated Genes (ISGs), which exhibit numerous functions such as targeting viral entry, RNA expression, protein synthesis, assembly or release through multiple mechanisms (89–91). For example, members of the IFN-inducible transmembrane (IFITM) family have the capacity of limiting viral entry and replication (92, 93). Another noticeable effect of IFN-γ is the induction of the OAS (oligoadenylate synthetase)-RNase L (latent ribonuclease L) pathway which functions to detect foreign RNA and to cleave both host and viral RNA (94). At the other end of the viral life cycle, Viperin (virus inhibitory protein, endoplasmic reticulum-associated, IFN-inducible) inhibits the virus release by blocking budding at the plasma membrane (95). Interestingly, Viperin acts in a similar manner as bisphosphonates, a class of drugs known to activate γδ T cells. Indeed, it inhibits farnesyl diphosphate synthase (FPPS), altering membrane fluidity by disrupting lipid rafts and interfering with virus budding as a consequence (96). Thus, one can hypothesize that administration of bisphosphonates for *in vivo* γδ T cells activation, as routinely done clinically (Cf. Part 4), will have a beneficial synergistic antiviral action.

γδ T cells produce a high amount of IFN-γ upon stimulation (97–100), commencing as early as 4 hours post-activation (101). Several studies show the central role of γδ T cell-secreted IFN-γ in the antiviral response (102–104). As an additional immunostimulatory mechanism, the high concentration of IFNs produced by infected cells and immune cells including γδ T cells themselves in inflamed areas (105) will reinforce activation of the immune cell pool, therefore augmenting the antiviral response (106).

Due to the evolutionary pressure of the anti-viral effects of IFN-γ, numerous strategies have arisen in viruses to subvert this protective mechanism. Other complementary and non-redundant mechanisms, such as TNF-α, which is also produced by the γδ T cell, are required. TCR triggering induces massive production of TNF-α by γδ T cells, as early as 20 minutes after stimulation (107, 108). The protective effect of TNF-α for antiviral immunity has been shown in a number of cases, such as infection by CMV (109), HSV (110) and vaccinia virus (111). In addition to its effect on infected cells, TNF-α is necessary for inducing resistance in uninfected cells, and for optimal activation of γδ T cells and their cytokine production. In this regard, TNF-α can act as a co-stimulatory signal for a sustained response to TCR triggering (112) which implies a positive feedback loop not dissimilar to the one observed with IFN-γ.

After activation *via* the TCR, even if the majority of γδ T cells were expressing only IFN-γ, the appearance of cells producing both IFN-γ and TNF-α has been noted (113), suggesting that different subsets with diverging antiviral functions might appear during activation, depending on the context (114). It is known that TNF-α and IFN-γ have a synergistic effect, providing a heightened antiviral function to the γδ T cells with the capacity to produce both (115). A diverse range of other cytokines including GM-CSF, IL-4, IL-5 and IL-8 are produced by γδ T cells following viral infection (116, 117), participating in the systemic immune response. Similar to other sentinel cells, γδ T cells also secrete chemokines such as CCL2, CCL3, CCL4, CCL5, and CCL22 to recruit pro-inflammatory effectors, accelerating the elimination of pathogens and the repair of damaged tissues (116, 118).

In addition to their direct anti-infection activities and their recruitment of other immune cells, γδ T cells help to establish the adaptive response by contributing to dendritic cell maturation (119–121) but also by acting as professional Antigen Presenting Cells (APC) themselves (122). Indeed, they can efficiently internalize, process and present pathogen-related antigens from both free viral particles (123) and infected cells (124) to other effector immune cells (125). These γδ-T APCs express approximately similar levels of the MHC-II antigen-presenting molecule HLA-DR and of the costimulatory molecules CD80/CD86 to conventional APCs such as dendritic cells, allowing an efficient induction of CD4+ αβ-T-cell responses (126). Moreover, γδ-T APCs’ ability for cross-presentation (a process describing the internalization of exogenous antigens and their degradation for peptide loading on MHC-I antigen-presenting molecules) allow them to equal or even exceed dendritic cells’ capacity to induce CD8+ αβ-T-cell proliferation and effector functions (126, 127). In addition to their capacity for antigen presentation, γδ-T APCs change their migratory properties during activation, including the expression of the chemokine receptor CCR7, allowing their homing to the draining lymph nodes where they can activate virus-specific αβ-T-cells (128).

Another role for γδ T cells in the initiation of adaptive immunity is their helper function for the B cell-mediated humoral immunity (129). Besides their role in antibody production, γδ T cells are also key players in antibody-dependent cell-mediated cytotoxicity (ADCC) *via* their expression of FcγRIII (CD16) (130, 131). Moreover, in the case of CMV infection, CD16 has been shown to be upregulated in γδ T cells (132) and implicated in viral inhibition *via* direct recognition of IgG-opsonized virions and stimulation of IFN-γ production (133). Interestingly, CD56 expression, upregulated upon stimulation (134) and associated with cytolytic effector functions in γδ T cells (135) might be only a marker of co-expression with CD16. Thus, the better observed antiviral activity of CD56+ γδ T cells would be essentially due to the CD16-mediated degranulation pathway (136).

The antiviral capacity of γδ T cells has been illustrated by different studies using a variety of *in vitro* infected cells. They highlight the relative importance of each pathway and their modulation depending on the infectious context. For example, in a model of influenza virus-infected A549 lung alveolar epithelial cell line, Li et al. have proven by targeted inhibition the reliance of γδ T cells on the perforin and Granzyme B pathway, as well as NKG2D, FasL, TRAIL and IFN-γ (116, 137). This cytotoxic profile was confirmed in different *in vitro* models, including EBV-infected B cell lines (23) and HIV-infected lymphocytes (58, 138).

*In vivo*, activated γδ T cells have also proven to efficiently clear human influenza virus in humanized mice models (139). In humans, a study in 205 renal allograft recipients showed that CMV infection directly precedes γδ T cell expansion, and is the only clinical parameter associated with this expansion (140). Importantly, CMV-infected patients who develop delayed γδ T cell expansion have a higher viral load, more symptoms and longer disease than patients with early expansion, showing another link between γδ T cells and viral infection (141). This resolution is likely to be dependent on TCR stimulation triggering the perforin-granzyme B pathway as well as the production of IFN-γ (142, 143). Both αβ and γδ T cells respond to viral infection, as in the case of EBV-induced mononucleosis, but only the latter keeps a high frequency during the convalescent phase, consistent with their immune surveillance role (65). In acute hepatitis B, peripheral γδ T cells are activated and exhibit increased cytotoxicity and capacity for viral clearance (144). There is a negative correlation between activated γδ T cells and clinical markers of hepatitis progression (145), and in chronically-infected patients there is a marked reduction in the proportion and cytotoxicity of circulating γδ T cells compared to healthy donors, this decreased antiviral function correlating with the persistence of HBV (146, 147). Early HIV infection is also associated with reduced number and function of γδ T cells in the blood and endocervix (148, 149). This loss is proportional to viremia (150, 151) and might be a contributing factor in the establishment of viral persistence in AIDS, notably by reducing the level of IFN-γ (152). Interestingly, this appears to precede the loss of CD4+ αβ T cells, the major target of HIV, suggesting that γδ T cell impairment is one of the very first immune failings during HIV infection (153). Moreover, HIV‐infected elite controllers have elevated levels of circulating γδ T cells compared with HIV‐negative controls or HIV‐infected individuals on antiretroviral therapy (154), highlighting again a link between γδ T cells and disease outcome. In this latter category of antiretroviral treated patients, a slow but steady reconstitution of the γδ T cell pool to near-normal levels is observed (155, 156). Combined treatment with zoledronate (a γδ T cell-stimulating drug) and Interleukin-2 (IL2) in HIV patients induced activation and expansion of their circulating γδ T cells, and a subsequent heightened immune response characterized by dendritic cell maturation and CD8+ T cells responses (157) showing the efficiency of such intervention.

### A Case Study of γδ T Cell Antiviral Function: Coronaviruses

To illustrate the points discussed above, the next part of this review will focus on the case of the SARS-CoV-2 virus, responsible for the 2020 pandemic, which has generated a worldwide effort and an unprecedented amount of data for a better understanding of viral infection and the immune response to it.

SARS-CoV-2 belongs to the betacoronavirus genus and causes a highly infectious respiratory disease called COVID-19. Its closest relative among human coronaviruses is SARS-CoV, with 79% genetic similarity (158). The pathophysiology of SARS-CoV-2 infection resembles that of SARS-CoV infection, with progression in some individuals to acute respiratory distress syndrome (ARDS) characterized by aggressive inflammatory responses in the lower airways and responsible for 28% of fatal COVID-19 cases. As such, severe COVID-19 is not only due to direct effects of the virus but also in part to a dysregulated immune response inflicting multi-organ damage, especially in the cardiac, hepatic and renal systems (159).

This immunopathology is defined by a suppression of the early pro-inflammatory response. Indeed, SARS-CoV-2 is able to inhibit several transcription factors pivotal for the antiviral response such as NF-kB and IRF3/7, resulting in limited IFN production and signaling, reduced recruitment of immune cells and viral evasion. This precipitates pathogenesis and mortality in susceptible individuals (160). Reports on severe COVID-19 patients also showed altered immune composition, with increased total neutrophils and reduced lymphocyte count in the peripheral blood (161), and a correlation between lymphocytopenia, serum IL-6 concentration (a hallmark of cytokine storm), and disease severity (162, 163). Moreover, as patients progress toward symptomatic stages, an increasing proportion of exhausted PD1+ and TIM3+ lymphocytes are seen, highlighting the failure of the adaptive system to control infection in these cases (164). COVID-19 is also characterized by its demographics, with a high susceptibility among older males (14.8% case fatality ratio after age 80 Vs 2.3% total; men roughly 1.5x more likely to die than women) (165, 166). Indeed, most children with COVID-19 are asymptomatic and have a normal lymphocyte count (167). One of the striking differences between young and elderly immunity is the strong innate responses observed in the former (168), leading to early control of infection at the site of entry. Multiple innate immunity aberrations have been reported in the elderly: desensitization of dendritic cells, reduced TLR responses, dysregulated IFN response, decreased macrophage and neutrophil function, reduced NK activity, and relevant to this discussion, decreased γδ T cell proliferation and number (169–171). It has also been observed that there is altered function and phenotype among circulating γδ T cell in the elderly, notably a lower response and a lack of memory cells (172–174). In women, this phenotypic change is not observed, and the γδ T cell reduction occurs later in life and is less pronounced than in men (175).

So innate immunity status and particularly γδ T cell function can shape the viral response and be a determinant of disease progression. Currently, only a few studies are available on the host innate immune response of COVID‐19 infected patients. It’s been shown that as the first line of defense, innate immunity must block the virus in the upper airways in the first 10-12 days from infection (5-7 from the disease onset) for an efficient resolution of the infection (176) and that it indeed performs with great efficiency in the majority of individuals (177). But in the case of the deleterious inflammation associated with severe COVID-19, a body of evidence suggest that it is due to a failure to activate the immune system during a critical early time window, and to a subsequent primary cytokine release syndrome triggered as a delayed emergency response to uncontrolled SARS-CoV-2 replication (178, 179). The priority therefore would be to promote an early and robust immune response for effective viral clearance and the prevention of symptomatic infection as well as viral transmission.

During the 2003 coronavirus outbreak, health care workers that survived SARS-CoV infection had a selective expansion of the blood Vδ2 γδ T cells, observed 3 months after the disease onset (180). No expansion of non-innate αβ T cells was detected at this timepoint. Interestingly, these γδ T cells were able to directly kill SARS-CoV infected target cells in an IFN-γ-dependent way, and their increase was proportional with anti-SARS-CoV IgG titers, suggesting their protective role during coronavirus infections.

There is currently a paucity of studies including the γδ T cells in their immune characterization of COVID-19, but the few studies that investigated this population gives us an interesting perspective on their role during the fight against SARS-CoV-2:

In accordance with the general lymphocytopenia, the percentage of γδ T cells in the blood of patients hospitalized for COVID-19 (on average 10 days after the onset of clinical symptoms) is lower than that of healthy controls (181, 182). Interestingly, there is a shift in γδ T cell phenotype during the 2 weeks of hospital admission, with a transition toward effector (memory) cells more capable of tissue infiltration, as confirmed by Odak et al. (183). The blood γδ T cell reduction is indeed associated with their recruitment in the airway tissues (184, 185). Moreover, γδ T cells’ level of stimulation (CD69 positivity) is increased in the blood compared to healthy controls and is even higher in the infected tissues than in the blood, showing their activation at the injury epicenter (186). Lei et al. (187) confirmed the γδ T cell activation in blood, with increasing expression of CD4 and CD25, and showed no sign of exhaustion as assessed by PD1 expression. The expansion of a CD16+ γδ T cell population in COVID-19 has been observed in single-cell transcriptional profiling of 13 patients. In the study, the presence of this CD16+ γδ T cells subset is strongly associated with moderate disease and almost absent in the severe condition (188). Another team comparing immune signatures between 63 COVID-19 patients and 55 Healthy Controls also confirmed the depletion of γδ T cells in the blood and showed that while the number of Vδ1 is not different from controls or between severity groups, the Vδ2 depletion is proportional to the disease severity (189). The authors then suggest that it could be used as a diagnostic or prognostic marker, a suggestion supported by Carissimo et al. who showed that a Neutrophil/Vδ2 ratio is a better prognostic marker of COVID-19 severity than the Neutrophil/CD8+ Lymphocytes ratio (190). They also showed that γδ T cells are generally activated, as seen by their upregulation of the activation marker CD38 and differentiate into central memory cells after recovery. Expansion of the γδ T cell pool has also been noted concomitantly of the remission phase in a single-cell analysis of 2 severe COVID-19 patients (191).

**Figure 1:**
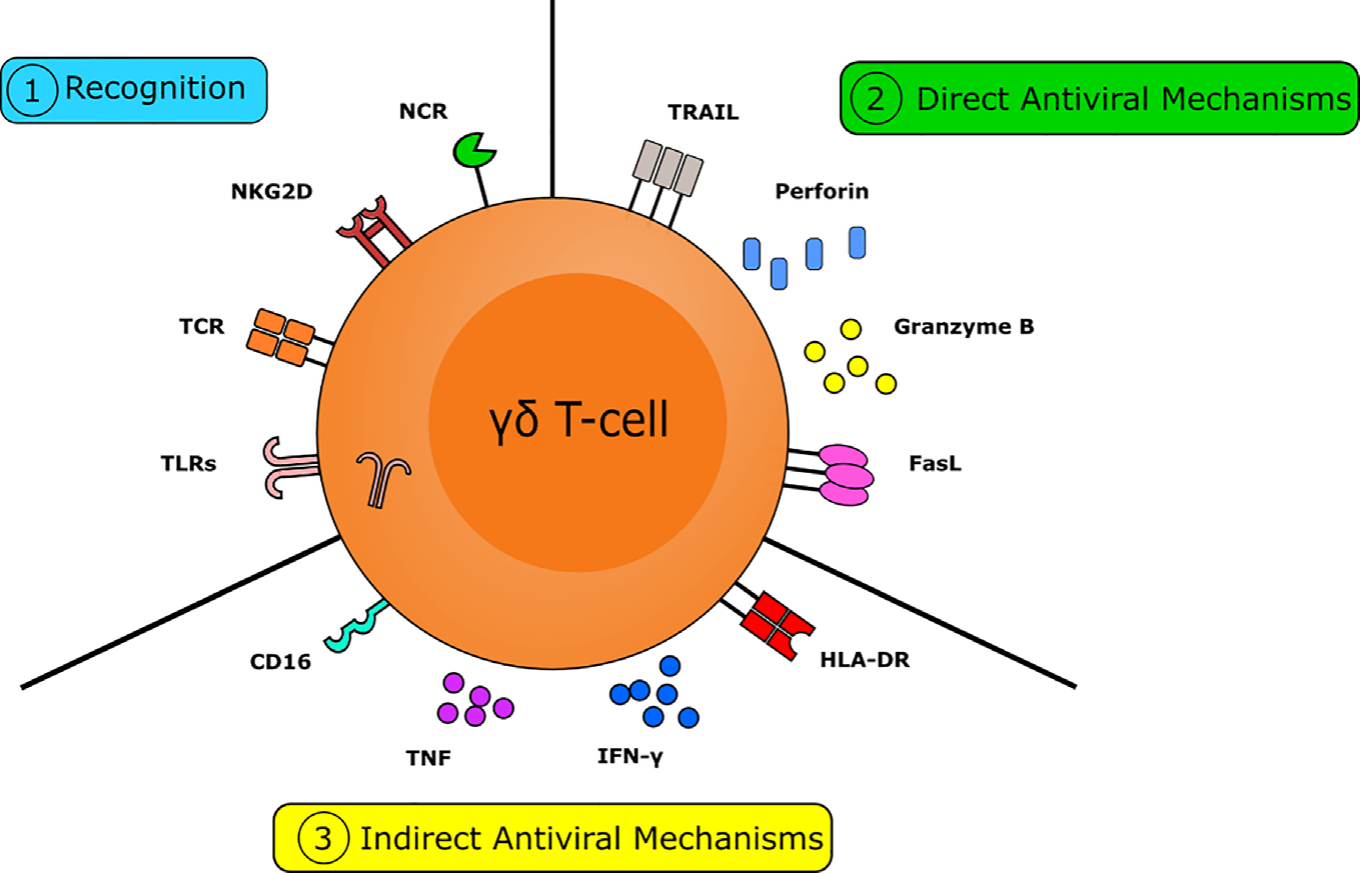
The multifactorial capacity for the γδ T-cell to interact with viruses and virally infected targets. Numerous pathways are crucial in the γδ T-cell mediated antiviral response. γδ T-cells are capable of rapidly recognizing virally infected cells. This can occur *via* the detection of isopentenyl pyrophosphate (IPP) by the T-cell receptor (TCR), *via* recognition of stress-induced molecules by NKG2D, or *via* the recognition of viral molecules and PAMPs by NK-type receptors and TLR, respectively. γδ T-cells have numerous mechanisms to directly combat viral infection. Direct antiviral mechanisms are mediated by cytolytic molecules, such as perforin and granzyme B, to induce cytolysis and by the expression of death receptors, including FasL and TRAIL, to induce apoptosis. γδ T-cells also have several indirect mechanisms capable of combatting viral infection. Indirect antiviral mechanisms are mediated by cytokines, such as IFNγ and TNF, by the expression of MHC-II allowing them to act as APC to direct the adaptive immune response and *via* expression of CD16 to trigger antibody-dependent cellular cytotoxicity. Together these actions make the γδ T-cell a crucial component in the immune response to viruses.

All the advantages highlighted above, including rapid activation, MHC independency, ability to traffic to infected tissues and potent antiviral function makes γδ T cells attractive candidates as therapeutic tools (192) (**Figure 1**). In the next section, we will focus on this therapeutic potential.

## The Art of War: γδ T Cell-Based Therapeutic Strategies

There are 2 major modalities for taking advantage of γδ T cell capabilities in a clinical context: *ex vivo* activation with a subsequent adoptive transfer, or direct *in vivo* activation.

### *Ex Vivo* Stimulation and Adoptive Cell Therapy

The *ex vivo* approach relies on γδ T cell isolation from Peripheral Blood Mononuclear Cells (PBMCs), *in vitro* stimulation with products such as bisphosphonates, pAg or monoclonal antibodies (193), and injection of the activated cells into patients (194). The safety and efficacy of this approach have long been proven in the treatment of cancers, with dozens of clinical trials involving isolation, expansion and adoptive transfer of up to 1x10^10^ γδ T cells (195).

This strategy is also implemented as antiviral therapy against various infections and has shown promising results. The first necessity for an optimal cell product is to stimulate γδ T cells in a way that maximizes their antiviral response. This has been achieved for example in a model of H1N1-infected macrophage, where γδ T cells expanded with isopentenyl pyrophosphate (IPP), a phosphoantigen, are able to effectively kill target cells and to inhibit viral replication, notably due to their high production of IFN-γ (116, 196). Similarly, when expanded with Pamidronate (PAM), a bisphosphonate, γδ T cells can also effectively kill influenza-infected lung alveolar epithelial cells *in vitro* thus inhibiting viral replication (137). These results have been confirmed in models of HCV as well as CMV infection (104, 197). Furthermore, Zoledronic Acid (ZA), another bisphosphonate, has been used *ex vivo* in PBMCs from HIV+ individuals and resulted in expansion of γδ T cells displaying cytotoxic capabilities and potent ADCC function, demonstrating that this protocol is able to reactivate effector functions in patient’s cells (198). PAM expanded cells from HIV-infected patients showed similar cytotoxicity against HIV-infected cells (199), illustrating that various avenues can be chosen to harness γδ T cells’ antiviral functions in a clinical setting.

The second step of this strategy involves the adoptive transfer of activated γδ T cells, which have been shown to be safe and effective in pre-clinical models of infectious disease. In mice infected with enterovirus or CMV, the adoptive transfer of γδ T cells was able to provoke a Th1-type response associated with viral control and better survival (200–202). In humanized mice infected by the influenza virus, injection of PAM-activated γδ T cells resulted in controlled viral replication and reduced disease severity and mortality (203).

Thus, γδ T cell-based adoptive cell therapies have the potential to be used as an allogeneic “off-the-shelf” antiviral product, akin to the strategies used for example with NK cells (https://clinicaltrials.gov/ct2/show/NCT04365101). Despite this potential, clinical efficacy has yet to be proven, and the logistical challenges that come with an *ex vivo* cell product may hinder the development of this specific strategy. Hence, directly stimulating a patient’s γδ T cells *in vivo* could appear more desirable.

### *In Vivo* Activation

The *in vivo* approach involves systemic stimulation and expansion of γδ T cells, usually by administration of bisphosphonates or pAg. It’s also used routinely for cancer treatment, with no severe adverse effects and an efficient *in vivo* expansion of IFN-γ+ Perforin+ effector γδ T cells (204, 205) associated with stable disease or partial remission (206).

The use of humanized mouse models has generated interesting data in influenza infection. *In vivo* activation with PAM resulted in accumulation of γδ T cells in lungs and fewer symptoms, associated with reduced lung inflammation, fewer cell infiltrates and decreased levels of mediators such as IL-6, TNF-α or IP-10 (203). This finding has been supported by others, who also describe a 3-fold increase of γδ T cells 2 days after treatment, and lower viral replication and mortality (139). Non-human primate models provide an alternative to humanized mice in the interrogation of *in vivo* γδ T cells responses. The pAg HMBPP ((E)-4-Hydroxy-3-Methyl-But-2-enyl Pyrophosphate), in combination with IL2, has been shown to cause expansion of circulating IFN-γ+ Perforin+ γδ T cells *in vivo*, and accumulation in the lungs lasting at least 3-4 months, long after circulating levels had returned to normal (207). In a similar study, γδ T cells accumulated in the lungs were able to protect from pulmonary lesions caused by Yersinia pestis infection (208). Finally, in a model of tuberculosis, IFN-γ+ Perforin+ γδ T cells accumulating in the lungs attenuated the lesions and stimulated a CD8+ T cell adaptive immune response (209). These findings are consistent with the paradigm that circulating γδ T cells can traffic to the lungs for homeostatic protection against tissue damage during infection, suggesting their potential as immunotherapeutics against a variety of pulmonary pathogens. In humans, administration of ZA with IL2 has been carried out in HIV‐infected, antiretroviral naïve patients and was associated with γδ T cell expansion, dendritic cell activation and increased HIV‐specific CD8+ T‐cell responses (210), suggesting that this strategy can be used to restore impaired immune response observed in AIDS (211).

The advantage of bisphosphonates such as ZA and PAM is that they are already clinically approved, inexpensive and relatively safe drugs (212). Moreover, in the context of viral infection, they might have an additive clinical benefit, as they’ve been shown not only to stimulate γδ T cells but also inhibit the protein prenylation pathway and the cholesterol synthesis, both required for virus assembly (113, 213). Taken together, these effects strengthen the argument for their use as antiviral agents.

Another known mechanism of *in vivo* γδ T cell activation is by microbial products like listeria, mycobacteria or salmonella-derived vaccines (214–216). Indeed, there is accumulating evidence that innate immunity, including γδ T cells, is boosted by specific vaccination in addition to targeted adaptive immunity (217). For example, the influenza vaccine is able to induce virus-specific γδ T cell expansion along with CD4+ and CD8+ T cells stimulation (218), and the differentiation of these γδ T cells into an effector/memory phenotype, with increased perforin expression (219). Vaccination in a model of Simian Immunodeficiency Virus (SIV) in macaques has been shown to block infection early at mucosal sites, and this protection was associated with expansion of γδ T cells and maturation of dendritic cells (220). In addition to their designed effects, vaccines have long been shown to protect beyond their target antigen through induction of innate immune mechanisms termed non-specific heterologous effects and trained immunity (221). Thus, certain adjuvants such as TLR agonists (222), as well as live vaccines like polio (223) or measles (224, 225) induce long-term cross-protection against various infections through epigenetic, transcriptional, and functional reprogramming of innate immune cells such as macrophages, NK cells or γδ T cells (226). This reprogramming results in enhanced activation, and ultimately protection against secondary infection, resembling immune memory (227, 228). The most well-studied inducer of trained immunity is the Bacillus Calmette–Guérin (BCG) vaccine (229). It is composed of a live attenuated strain of Mycobacterium bovis originally given to young children to protect against tuberculosis, but recent studies demonstrated that its administration more broadly reduced mortalities from infectious diseases over the neonatal period (230, 231). It has then been postulated that the relative protection from COVID-19 reported in children might be attributed to their frequent vaccinations, and indeed some correlations between BCG vaccination policies and reduced infection and mortality rates due to SARS-CoV-2 have been reported (232–235). Indeed, even after correcting for many socioeconomic and pandemic-related confounders, data shows that for every 10% increase in the BCG index (degree of national universal vaccination), there is a 10.4% reduction in COVID-19 mortality (236). These results are still under debate (237) but have initiated numerous studies and clinical trials investigating the effect of BCG on nonspecific protection against SARS-CoV-2 infection or its severity (238–240) (https://clinicaltrials.gov/ct2/show/NCT04369794, NCT04362124, NCT04379336, NCT04350931, NCT04327206, NCT04373291, NCT04328441, NCT04348370). This non-specific protection could be harnessed independently of age, as a randomized controlled trial in elderly (60–75 years old) who received BCG vaccinations, showed a reduction of the incidence of acute upper respiratory tract infection (241). It has also been proven to protect against a variety of viruses like yellow fever, influenza, papillomavirus (HPV), Respiratory syncytial virus (RSV) or HSV (242, 243).

As a key cell type in the innate immune response, it is clear γδ T cells also play a role in contributing to trained immunity. Many studies have documented expansion of the γδ T cell population following vaccination with BCG, with these cells being one of the key producers of IFN-y in immunized children (244–246). Mycobacteria stimulation also induces γδ T cell cytotoxicity toward virus-infected cells (HSV and vaccinia), typical of the heterologous effect observed in trained immunity (247). Moreover, γδ T cells expanded after viral infection or BCG stimulation, differentiate into effector memory cells capable of a faster and more efficient response to a second infection (248–251). So BCG can be used to expand cytotoxic γδ T cells capable of eventually differentiating in long-lived memory cells allowing enhanced protection against subsequent infections.

The contribution of γδ T cells to the regression of BCG-treated melanoma patients has already been proven (252), and highlights the clinical potential suggested above for a similar setting in treatments of viral infections. Thus, BCG or its derivatives (253, 254) are attractive candidates for establishing trained immunity and stimulating early clearance of subsequent viral infection (255). Integrating innate immunity stimulation in the design of vaccines would also be a way of harnessing this under-considered potential (256). Indeed, by the choice of delivery route (257, 258) or adjuvant (259), one could balance the immune response to allow for complementary protection in instances where the adaptive immunity is failing. BCG itself could be used as an adjuvant or in a prime-boost strategy, as it has been shown to orient toward an antiviral Th1-type response and to enhance vaccine efficiency (260).

## Discussion

As highlighted here, the varied characteristics of γδ T cells support their role in controlling viral diseases in general and COVID-19 in particular. Considering the accumulating evidence on their multiple antiviral functions and their capacity to react early and to quickly prevent viral spread, we’re advocating for better inclusion of γδ T cells in the therapeutic armamentarium against viral infections. For example, a cheap and effective way of harnessing anti-viral innate immunity such as that mediated by γδ T cells would be to vaccinate the population with BCG in cases where there is no access to a specific vaccine, or as a supplementary boost to it, and the ongoing clinical trials using these strategies will be of tremendous importance for the optimization of γδ T cell-based therapies against viruses.

## Author Contributions

JC designed, wrote, and revised the manuscript. LR wrote and revised the manuscript. MB-S revised and edited the manuscript. All authors contributed to the article and approved the submitted version.

## Funding

This work was supported by the Institute for Cancer Vaccines and Immunotherapy (Registered Charity Number 1080343).

## Conflict of Interest

The authors declare that the research was conducted in the absence of any commercial or financial relationships that could be construed as a potential conflict of interest.
